# The outcomes of proximal femur replacement with hemiarthroplasty after tumor resection in individuals with Parkinson's disease

**DOI:** 10.3389/fsurg.2023.1279813

**Published:** 2023-10-13

**Authors:** Zhiqing Zhao, Jichuan Wang, Yu Guo, Jianfang Niu, Wei Guo, Rongli Yang, Taiqiang Yan, Xiaodong Tang

**Affiliations:** Musculoskeletal Tumor Center, Peking University People’s Hospital, Beijing, China

**Keywords:** proximal femur replacement, hemiarthroplasty, Parkinson’s disease, bone tumor, prosthesis

## Abstract

**Background:**

Patients with neurological disorders often experience a high incidence of postoperative complications following proximal femur replacement (PFR) surgery. The orthopaedist faces a significant difficulty in treating Parkinson's disease (PD) because of the weakened bone condition, excessive muscle tension, and increased risk of fractures. The objective of this research is to assess the impact of PD on PFR following tumor removal.

**Methods:**

A retrospective study was conducted from 2010 to 2020, focusing on a solitary institution, analyzing 9 patients diagnosed with PD who underwent PFR with hemiarthroplasty as a result of tumor removal. The study consists of 2 men and 7 women, with an average age of 71 (SD, 12) years. We assessed the outcomes after surgery in terms of pain management, quality of life, functional ability, occurrence of complications, and survival durations.

**Results:**

All nine patients underwent planned surgeries. Intraoperative complications was not observed. The average length of the follow-up period was 24 (SD, 20) months, ranging from 8 to 72 months. Despite the fact that 8 patients passed away due to tumor progression, the endoprostheses were still well at that point. The preoperative VAS score of 7 (SD, 1.87) decreased to a postoperative score of 2 (SD, 1.32). The KPS was improved to73 (SD, 7) from 52 (SD, 14), postoperatively. Post-surgery, there were notable enhancements in both pain levels and the overall quality of life scores. Following the surgical procedure, individuals are able to ambulate steadily, resuming their regular daily routines. Living patients had an average MSTS score of 21 (SD, 2.5), ranging from 17 to 25. In total, there were four (44.4%) patients suffered complications after surgery, comprising of one wound dehiscence, one prosthetic fracture, one hip dislocation, and one local recurrence.

**Conclusions:**

Significant improvements in function and pain relief can be achieved through PFR with hemiarthroplasty following tumor removal in patients with PD. The implementation of thorough preparation and carefull nursing results in reduced complications and improved outcomes in PD patients.

## Introduction

Primary and metastatic bone tumors frequently occur in the proximal femur (1). After advancements in adjuvant treatment and surgical methods, limb preserving surgery has become the primary approach to therapy, with no negative impact on survival rates (2–4). Large bone defect and loss of soft tissue can occur as a result of surgically removing the upper femoral segment. Modular implants are commonly utilized for bone defect reconstruction following tumor removal, resulting in favorable clinical results (5–7). The risk of hip dislocation after modular proximal femoral replacement (PFR) with hip arthroplasty, especially following tumor resection, cannot be ignored. According to recent research, prosthetic reconstruction has been found to have dislocation rates of up to 20% (5, 8–12). Several factors, such as the surgical method, condition of the abductor muscles, previous hip surgeries, and placement of the implant components (11, 13), are recognized as known causes that affect the dislocation rate.

Parkinson's disease (PD) is a degenerative disorder of the nervous system that progresses over time and presents various characteristics, such as fixed tremor, cognitive decline, reduced movement, decreased bone strength, and abnormalities in posture and walking (14). This disease is the second most prevalent neuromuscular disorder, impacting 150–200 individuals per 100,000 and 1% of the population aged 60 years and above (15). Among those patients, some will suffered bone tumors, including primary or secondary tumors. Orthopaedists find it stressful to deal with PD patients who have increased muscle tension, a greater likelihood of fractures, and compromised bone strength. Previous studies (16–19) have documented a significant mortality rate during the perioperative period, as well as early surgical failure and increased postoperative complications in hip fracture surgery for individuals with PD. Nevertheless, there is a lack of substantial evidence concerning PFR following tumor removal in PD patients. The objective of this retrospective analysis was to assess the medical results concerning individuals with PD who underwent PFR with hemiarthroplasty surgery as a result of tumor removal.

## Methods

A review was conducted on patients who underwent PFR with hemiarthroplasty due to bone tumors at a solitary institution between 2010 and 2020. The institutional review board in our hospital granted approval for this retrospective cohort study.

### Inclusion and exclusion criteria

In order to be included, patients needed to meet the following criteria: (1) having a pre-existing diagnosis of PD; (2) being diagnosed with either a primary malignant tumor or a metastatic tumor in the proximal femur; (3) undergoing reconstruction of the PFR and hip arthroplasty; and (4) being followed up for a period of more than 3 months. Patients who met any of the following criteria were excluded from this study: (1) presence of multiple metastases, (2) expected survival time of less than 3 months, (3) perioperative death, or (4) lost follow-up.

### Patients

From 2010 to 2020, our center provided treatment for 13 individuals diagnosed with PD through the use of PFR and hip replacement surgery following tumor removal. Incomplete follow-up data resulted in the exclusion of four patients from this study. Out of the 9 patients who were left, 2 were males while 7 were females, and their average age during the surgery was 71 (SD, 12) years, ranging from 55 to 86 years. Out of these patients, 33.3% were diagnosed with a primary malignant tumor, including one case of undifferentiated sarcoma, one case of fibrosarcoma, and one case of chondrosarcomas. Five patients had isolated proximal femoral bone metastase (2 lung carcinoma, 1 breast carcinoma, 1 renal carcinoma, and 1 stomach carcinoma). One patients had myeloma.

According to the categorization documented by Yang and colleagues (20). Two were classified as type I (located in the femoral head and neck), 4 as type II (intertrochanteric), and 3 as type III (under trochanteric). This cohort did not exhibit Type IV (proximal femur and ipsilateral acetabulum). Out of the total, seven were classified as A without any pathological fracture, while two were classified as B with a pathological fracture occurring.

### Preoperative preparation

All patients underwent preoperative imaging, which included x-ray film, computed tomography (CT), and magnetic resonance imaging (MRI) of the affected area. Orthopaedists carefully examined the MRI to ensure the removal of bone with tumor-free margins. The prostheses utilized in this research was manufactured by Lidakang (LDK Corp., Beijing, China). Moreover, the LARS® ligament (Laboratoire d'Application et de Recherche Scientifique, located in Arc-sur-Tille, France) was utilized for the purpose of soft tissue reconstruction.

### Surgical treatment

Every patient underwent the administration of general anesthesia. Next, individuals were positioned on their side with a slight bend in the hip.

**Figure 1:**
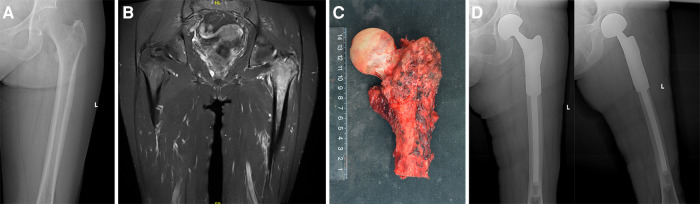
(**A**) The x-ray film before surgery that did not show significant abnormality; (**B**) the MRI showed a lesion in the proximal femur; (**C**) the excised specimen showing the femoral osteotomy was performed at least 2 cm beneath the tumor's border; (**D**) radiograph after surgery.

The Watson Jones approach was utilized for all procedures. At the same time, the tumor was also removed along with the puncture channel. The tumor was removed with a wide or marginal surgical margin while keeping the femoral nerve and sciatic nerve intact. We tried to preserve the gluteus medius, iliopsoas, and gluteus maximus to the greatest extent possible. The operation procedures are performed in the following manner: (1) The hip joint capsule is opened, and in certain cases of fractures, the capsule within the surgical area is removed. To achieve a negative margin, the osteotomy level was positioned at a minimum of 2 cm beneath the tumor's border (Figure 1). Afterwards, the femoral head, neck, and trochanter underwent surgical removal. Verify the measurements of the removed portion of the femur and the artificial joint. During the implantation of the prosthesis, the distal extremity was secured using bone cement, while the femur's neck was tilted forward by approximately 10°–15°. All participants in this research were provided with the synthetic ligament in order to attain a specific level of stability in the joint. For the reconstruction of the joint capsule and surrounding soft tissues, multiple nonabsorbable sutures were used to tightly wrap the LARS® ligament around the prosthesis in a spiral manner. In the end, the injury was cleansed and a tube for draining was inserted. After the surgery, the impacted limb was positioned in a neutral abduction stance.

Quadriceps femoris muscle exercise was started on the second day after surgery. Patients were permitted to bear weight as tolerated, but were advised to utilize a walker for a period of 3 months. This was done in an attempt to reduce stress on the abductors and subsequently minimize the need for abductor repair. Following this duration, individuals were gradually taken off their supportive equipment and given recommendations for muscle-building workouts.

### Data collection and follow up

Demographic data before surgery, surgical details (amount of bleeding during surgery and duration of operation), and postoperative information (oncological outcome, functional outcome, and complications) were documented.

All patients were monitored every 3 months for a duration of 2 years. To detect any indications of loosening, fracture, or local recurrence, all patients underwent both local x-ray imaging and CT scans. Pain level was evaluated using the visual analog scale (VAS). To evaluate the quality of life, Karnofsky performance scores (KPS) were documented, where a lower score indicates a decline in health status. The postoperative functional condition was assessed using the Musculoskeletal Tumor Society (MSTS) scale (21), which consists of six domains: pain, function, satisfaction, assistance, ambulation, and lower limb gait. A better function is indicated by a higher score.

## Statistical analysis

The statistical analyses were conducted using SPSS software, version 22.0. The mean and standard deviation (SD) was used to express all values. Paired *t*-test was used to compare pre- and post-operative VAS, KPS, and functional scores. Kaplan–Meier curves were created, with the date of the surgical procedure as the initial point, and death or the most recent contact date as the point of censoring. A significance level of less than 0.05 was attributed to the *P-*value.

## Results

### Surgical data

Planned surgeries were performed on all the patients. No deaths occurred during the surgery. The resection length of proximal femur was 14 (SD, 1) cm. The average duration of the operation was 216 (SD, 40) minutes, with a range of 170–300 min. The average amount of blood lost was 594 (SD, 188) mL, with a range of 300–900 ml. There were no significant complications during the surgery.

### Oncological outcome

**Figure 2:**
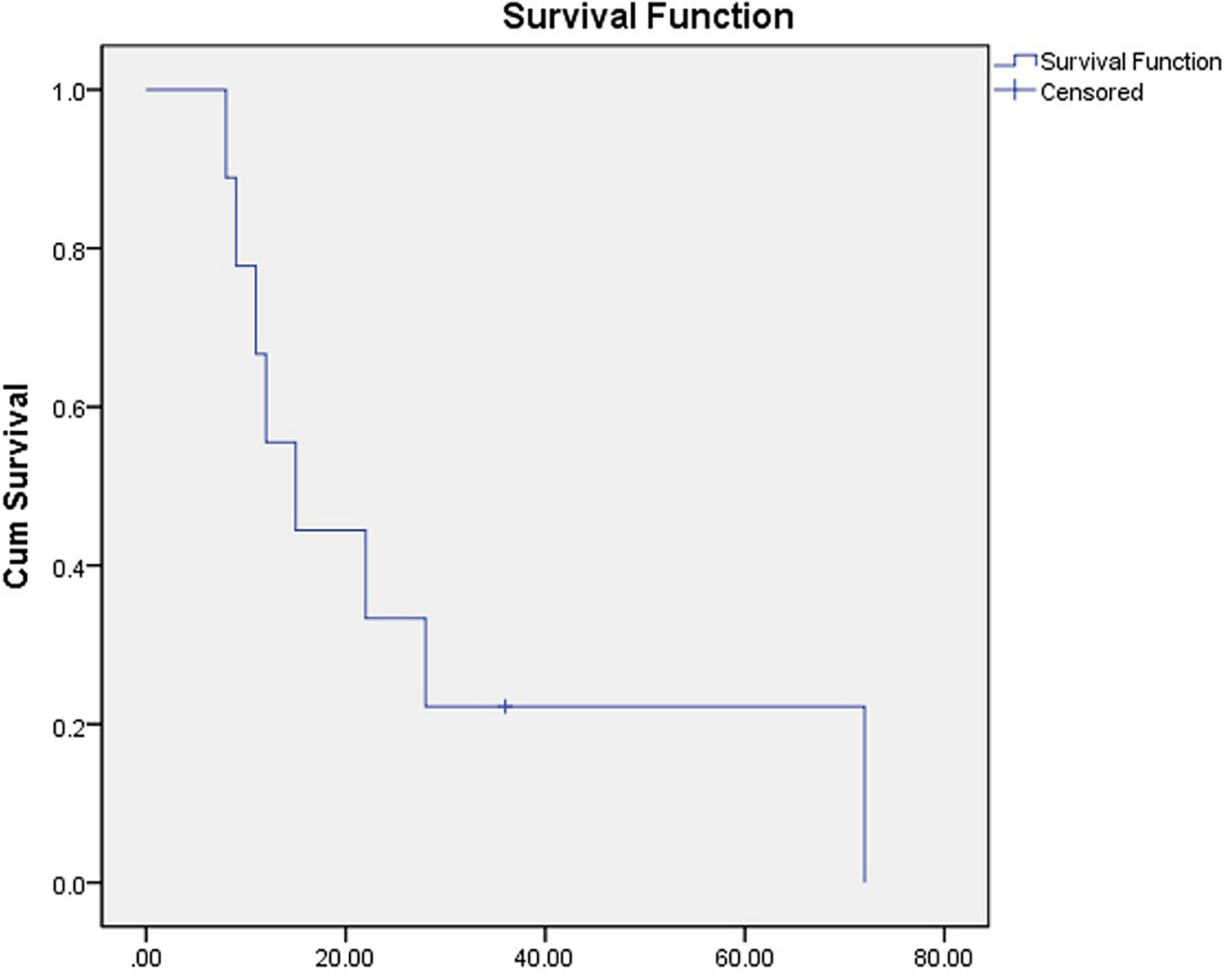
The curve of K-M survival.

Follow-up began at the time of the surgical procedure, concluding at the final office appointment or expiration. There were no patients who were not followed up with. Table 1 summarizes the demographic information, intraoperative details, and postoperative results. The mean duration of follow-up was 24 (SD, 20) months, ranging from 8 to 72 months. Despite the fact that 8 patients died due to tumor progression, the endoprostheses were operating efficiently at that point. According to Figure 2, the K-M survival estimate one year after the operation was 22%.

**Table 1:** Demographic, surgical data, and postoperative outcomes.

No.	Age/Sex	Fracture	VAS-preop	KPS-preop	VAS-post	KPS-post	Operation time (min)	Blood loss (ml)	MSTS	Followup time (month)	End
1	83/F	No	7	60	3	80	200	700	23	72	DOD
2	55/M	Yes	9	40	1	70	260	550	20	9	DOD
3	55/F	No	6	50	2	70	170	600	19	36	NED
4	63/F	No	9	40	2	60	190	400	21	11	DOD
5	82/M	No	9	50	5	70	200	500	20	12	DOD
6	76/F	No	4	70	1	80	190	900	24	22	DOD
7	86/F	No	5	60	1	80	210	300	17	15	DOD
8	75/F	Yes	8	30	2	70	300	800	20	8	DOD
9	65/F	No	6	70	1	80	220	600	25	28	DOD

### Postoperative outcomes

After the surgery, every patient demonstrated a decrease in pain level. The preoperative VAS score of 7 (SD, 1.87) decreased to a postoperative score of 2 (SD, 1.32). After treatment, there was a noticeable enhancement in the quality of life. The KPS was improved to 73 (SD, 7) from 52 (SD, 14), postoperatively. The analysis of paired *t*-test revealed a notable enhancement in pain and quality of life (*P *< 0.05).

### Functional outcome

Three months after surgery, we evaluated the affected limb functiona of all patients. They were able to walk steadily without experiencing any discomfort or slight pain, and have resumed their regular daily activities. Following PFR, the hip's range of motion (ROM) for flexion measured 65° (SD, 14°; range, 40°–80°), while abduction measured 19° (SD, 4°; range, 15°–25°). Living patients had an average MSTS score of 21 (SD, 2.5; range 17–25).

### Complications

In total, there were four (4/9, 44.4%) complications happened after surgery, comprising of one occurrence of wound complication, one prosthetic fracture, one hip dislocation, and one local recurrence.

**Figure 3:**
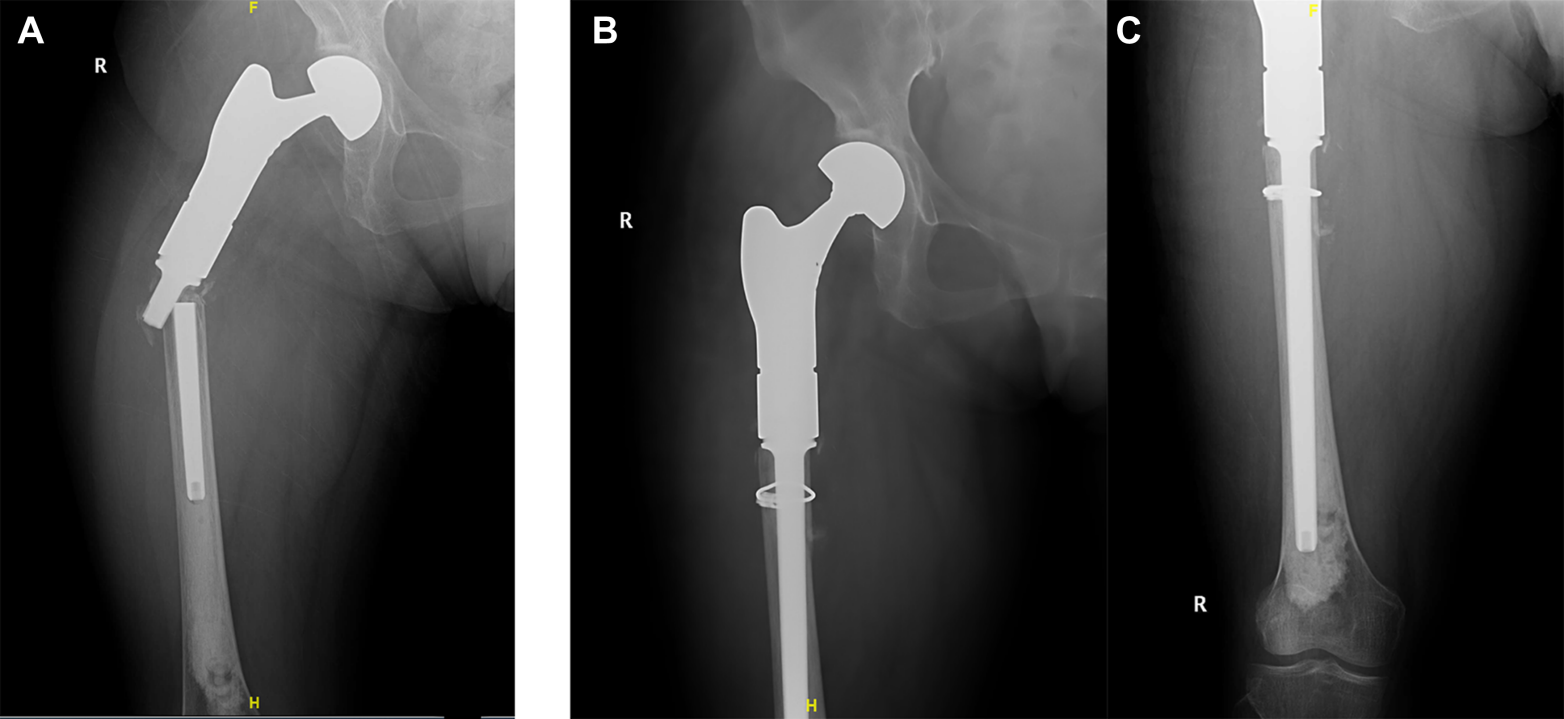
(**A**) The x-ray film showed breakage of femoral stem at 12 months postoperatively; (**B,C**) revision surgery was performed and prosthesis was replaced by a new and longer femoral stem.

Wound dehiscence was observed in one 82 years old patient. It was successfully managed by wound dressing and antibiotics. An accidental injury caused a fracture of the femoral stem in a 76 years old woman (Figure 3). Nevertheless, the x-rays did not indicate any signs of femoral stem loosening. After undergoing a surgical revision, a new longer femoral stem was implanted. One case with dislocation underwent closed reduction. Furthermore, a single patient exhibited a recurrence of the soft tissue in the local area throughout the duration of the post-treatment monitoring. Afterwards, the recurrent tumor was surgically removed.

## Discussion

Performing surgery on malignant tumors situated in the proximal femoral region can alleviate pain, restore functionality to the affected limb, and minimize complications resulting from prolonged bed rest. Consequently, the surgery prolongs the survival of those patients and improving quality of life (22, 23). One advantage of the PFR is its ability to directly repair the bone defect following tumor removal. This treatment is particularly appropriate for individuals experiencing significant loss of bone mass in the proximal femur and having a weak overall or local health condition. This study on feasibility discovered that performing PFR after removing tumors in patients with PD is advantageous in attaining immediate and long-lasting pain alleviation, enhancing quality of life and functional results.

Previous research has shown that the outcomes of hip arthroplasty in individuals with PD have been unsatisfactory due to the prevalence of medical complications resulting in high rates of morbidity and mortality (17, 24). Several research studies have documented a significant mortality rate during the initial period following surgery, reaching as high as 47% within six months (25). Turcotte et al. conducted a comparative study looking back at past events. They find that PD patients with hip fractures treated by hemiarthroplasty have worse results than those treated by internal fixation, indicating that hip arthroplasty might not be recommended for PD patients. According to reports, the death rate among PD patients who undergo surgery for proximal femur fractures is similar to that of the overall population. However, these patients experience greater morbidity (18). Although the surgical outcome in PD patients is poor, the PFR with hip arthroplasty is still essential for those with malignant tumors of proximal femur. What we should do is to find solutions to decrease the complication rate.

The study demonstrated that the postoperative results in patients with PD are satisfactory. It is important to maintain the belief that effective management of PD results in reduced complications and improved results (17, 26). Typically, parkinson's disease occurs between the ages of 40 and 70 years, with a greater frequency in older individuals. One of the common complication after surgery was wound healing problem. The main cause of inadequate wound healing is frequently the deteriorated overall health of elderly individuals. These individuals consistently exhibit reduced regenerative capacity, susceptibility to adverse nitrogen imbalance, compromised immune response, low tolerance for blood loss, history of chemotherapy or radiotherapy, and multiple surgical procedures. Additional complications that were reported included damage to the sciatic nerve, blood clot formation and blockage, dislocation of the hip joint, loosening of the artificial joint, and fractures. According to additional research, patients with primary bone tumors who are expected to live longer are more likely to experience higher rates of aseptic loosening and periprosthesis fractures (27, 28). There were no instances of prosthesis loosening observed during the follow-up period, although this could potentially be linked to decreased survival rates and reduced mobility in patients with metastatic tumors. During the follow-up, there was only one hip dislocation recorded, which could possibly be attributed to the extensive removal of the joint capsule in this particular study. Furthermore, removing the muscles along with the tumor will significantly modify the anatomical framework, leading to a notable impact on the joints' dynamic stability and ultimately causing the occurrence of hip dislocation (28, 29).

What factors should be considered when deciding between total hip arthroplasty (THA) and hemiarthroplasty? The condition of the acetabular cartilage, the age of the patient, and their life expectancy (30) all have an impact on it. The occurrence of dislocation is more frequent following THA compared to hemiarthroplasty, with a rate that is five times higher (31). Hemiarthroplasty provides satisfactory durability for the majority of patients with malignant tumors and limited life expectancy, excluding those with type IV tumors. None of the 9 patients in our research had any involvement of the ipsilateral acetabulum. As a result, hip hemiarthroplasty was carried out, resulting in reduced operating time, decreased risk of infection, and minimized patient discomfort and trauma.

The study utilized the cemented femoral stem as it is better suited for patients with bone metastasis. The appropriateness arises from the prompt stabilization and optimal advantage during the restricted duration of these individuals' lives. Previous research has indicated that the cemented type exhibits a lower Harris hip score compared to the non-cemented type; however, it also demonstrates a lower postoperative fracture incidence rate (32). In the area of joint replacement surgery, the cemented variety exhibits reduced complications and lower rates of revision (33, 34), along with a decreased occurrence of infection or aseptic loosening (29, 35). It was proposed by Mathew et al. (18) that THA can be performed in patients with PD, as long as there is a cautious assessment of neurological findings and appropriate indications. Our preference is for cemented bipolar arthroplasties, and we adhere to the methodology outlined by Miyamoto et al. (36). According to the individual who claimed that bone cement is secure and suitable for elderly individuals. In this research, we observed no instances of patients experiencing loosening of the femoral stem, which is deemed satisfactory.

It is worth mentioning that individuals with neurological conditions have a higher likelihood of encountering postoperative complications such as surgical site infection, urinary tract infection, and respiratory infection (37). We also will face the same problem in PD patients undergoing PFR. Therefore, individuals with PD require increased and thorough medical attention in order to reduce both specific and overall complications. The treatment of PD in a medical setting can greatly enhance symptoms and has led to an increase in the lifespan of patients (16). Thanks to advancements in healthcare, individuals diagnosed with PD can now enjoy extended lifespans and lead more vibrant, productive, and independent lives. We also found that with thorough preparation and careful nursing, postoperative pulmonary infection could be prevented. If the life expectancy of PD patients with malignant tumor is less than 3 months, their organs are less able to tolerate surgery. Conservative treatment or palliative surgery is recommended for these patients as they are at a higher risk of developing pulmonary infection. It was determined by Johnson et al. (30) that addressing neutropenia and anemia before surgery, providing supplements, and temporarily stopping chemotherapy can be beneficial in these situations. In addition, it is important to take into account the management of pain, engaging in early functional exercise, and using antibiotics in a rational manner. In other cases, the surgery itself does not directly contribute to mortality, but rather, complications arising from prolonged bed rest are the primary cause of death. Intraoperative complications include hypotension, oxygen desaturation, embolism, and cardiac arrest, but these rarely lead to death. Instead, the main reasons for death (27) are postponed additional treatment and the failure of prostheses caused by infections after surgery, while deaths caused by cardiopulmonary complications after surgery make up 1%–10% (31).

Due to the increased difficulty of performing joint PFR in elderly patients with PD, their surgical criteria are more rigorous compared to younger patients. When the patient has a pathological fracture or an imminent fracture with severe dysfunction (Mirels score >9) (38), this surgery should be considered. After the surgery, we observed significant improvements in the postoperative VAS and KPS scores when compared to the scores before the surgery. The results showed that patients with PD who received PFR experienced a reduction in pain, an enhancement in their quality of life, and an improvement in their overall functioning.

There were certain constraints in this research. Initially, there was a restriction on the number of cases, and furthermore, patients had diverse pathological diagnoses, potentially impacting their duration of survival.

## Conclusion

Our study findings indicate that individuals with proximal femoral malignant tumors who have PD experience positive outcomes from undergoing PFR and hemiarthroplasty. These procedures effectively alleviate pain, enhance quality of life, and result in satisfactory functional improvements. The implementation of thorough preparation and careful nursing results in reduced complications and improved outcomes.

## Data Availability

The original contributions presented in the study are included in the article/Supplementary Material, further inquiries can be directed to the corresponding author.
